# Supplementary figures and images for: Metabolomic profiling identifies complex lipid species and amino acid analogues associated with response to weight loss interventions
Source: PLoS One. 2021 May 27;16(5):e0240764. doi: 10.1371/journal.pone.0240764 (PMC8158886; doi:10.1371/journal.pone.0240764)

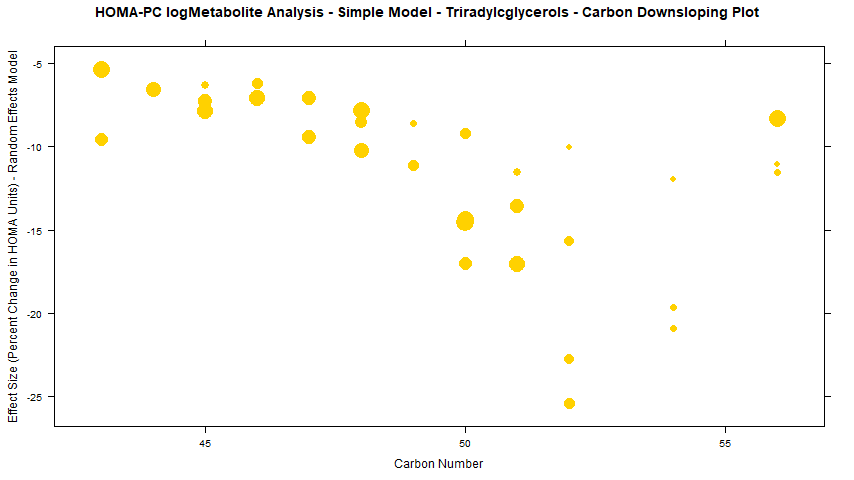

Supplement: S1 Fig — Plot of TAG metabolite effect size from random effects meta-analysis of univariate model vs number of carbon atom in the TAG. (PNG) [file pone.0240764.s005.png]

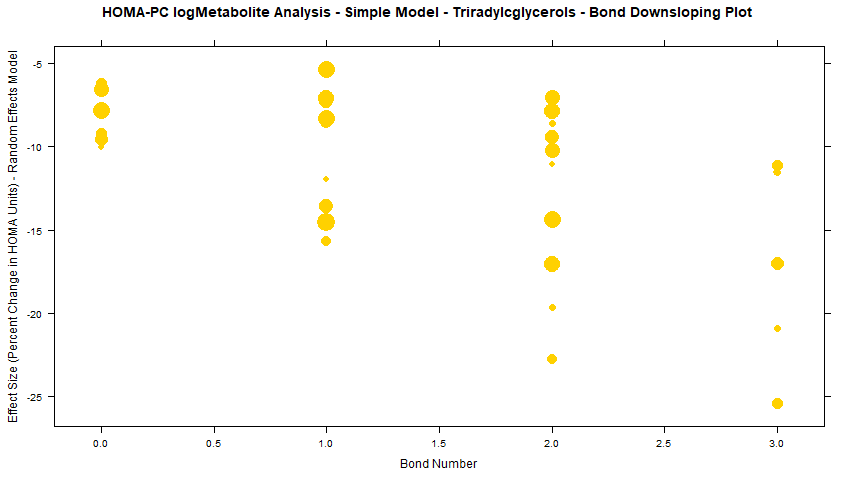

Supplement: S2 Fig — Plot of TAG metabolite effect size from random effects meta-analysis of univariate model vs number of double bonds in the TAG. (PNG) [file pone.0240764.s006.png]
